# Population genetics provides new insights into biomarker prevalence in dab (*Limanda limanda* L.): a key marine biomonitoring species

**DOI:** 10.1111/eva.12074

**Published:** 2013-05-23

**Authors:** Niklas Tysklind, Martin I Taylor, Brett P Lyons, Freya Goodsir, Ian D McCarthy, Gary R Carvalho

**Affiliations:** 1 Molecular Ecology and Fisheries Genetics Laboratory, Environment Centre Wales, School of Biological Sciences, Bangor UniversityGwynedd, UK; 2 School of Biological Sciences, University of East AngliaNorwich, UK; 3 Weymouth Cefas LaboratoryWeymouth, UK; 4 Lowestoft Cefas LaboratoryLowestoft, UK; 5 School of Ocean Sciences, Bangor UniversityMenai Bridge, UK

**Keywords:** biomonitoring, disease biology, ecotoxicology, fish, microsatellite, population genetics, random forest, temporal genetic stability

## Abstract

Bioindicators are species for which some quantifiable aspect of its biology, a biomarker, is assumed to be sensitive to ecosystem health. However, there is frequently a lack of information on the underlying genetic and environmental drivers shaping the spatiotemporal variance in prevalence of the biomarkers employed. Here, we explore the relative role of potential variables influencing the spatiotemporal prevalence of biomarkers in dab, *Limanda limanda*, a species used as a bioindicator of marine contaminants. Firstly, the spatiotemporal genetic structure of dab around UK waters (39 samples across 15 sites for four years: 2005–2008) is evaluated with 16 microsatellites. Two temporally stable groups are identified corresponding to the North and Irish Seas (average between basin 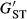 = 0.007; 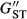 = 0.022). Secondly, we examine the association between biomarker prevalence and several variables, including genetic structuring, age and contaminant exposure. Genetic structure had significant interactive effects, together with age and some contaminants, in the prevalence of some of the biomarkers considered, namely hyperpigmentation and liver lesions. The integration of these data sets enhanced our understanding of the relationship between biomarker prevalence, exposure to contaminants and population-specific response, thereby yielding more informative predictive models of response and prospects for environmental remediation.

## Introduction

Human activities often result in the transformation or pollution of habitats with high levels of contaminants. Aquatic environments are particularly threatened as they often serve as intentional or unintentional repositories of chemical contaminants (Islam and Tanaka 2004). Such anthropogenic effluents may concentrate in lakes, estuaries, coastal waters and ultimately oceans, where high levels may be found (Scheringer 2009). Heavily polluted environments may have deleterious effects on wildlife, ecosystems and ultimately, on ecosystem services (Tanabe 1988). Governments in many countries are now legally bound to monitor levels of contaminants in aquatic environments; for instance, the UK's aim for ‘clean, healthy, safe, productive and biologically diverse ocean and seas’ has resulted in the production of a UK Government Charting Progress 2 report (Defra 2010). At a wider European level, the OSPAR convention (OSPAR Commission 2000) and the European Union Marine Strategy Framework Directive (European Parliament 2008) promote coordination of environment management, including management of chemical contamination, among member states. To monitor contaminants and its effects on biota, diverse tools and approaches for detection and remediation of anthropogenic impacts have been developed. One such approach is the use of bioindicator species for which parameters such as presence or absence, population size, health status and other proxies of ecosystem health (biomarkers) are recorded regularly, thereby providing time series data (Phillips and Segar 1986; Whitfield and Elliot 2002; van der Oost et al. 2003). Changes in such parameters can be analysed in conjunction with variation in contaminant levels and other environmental variables, to assess the impact of contaminants on natural ecosystems, their trends over time and the effectiveness of environmental policies (Vethaak et al. 2009; Lyons et al. 2010).

Due to their benthic lifestyle and close proximity to settled contaminants, flatfish are valuable for bioassessing pollution in estuaries and coastal waters (Köhler et al. 1992; Stentiford et al. 2009). Their medium to high trophic status renders them prone to biomagnification of contaminants (Bragigand et al. 2006). Moreover, long lifespan (Deniel 1990) permits the development of long-term diseases (Stentiford et al. 2009; Vethaak et al. 2009), which also enhances their value in biomonitoring schemes. Accordingly, dab (*Limanda limanda*) is a key European bioindicator sentinel species (Feist et al. 2004; Stentiford et al. 2009; Vethaak et al. 2009) and is employed in biomonitoring programmes such as the Joint Assessment and Monitoring Program, JAMP (OSPAR 2009), and the Clean Seas Environmental Monitoring Programme, CSEMP (Cefas 2003). Detailed biomonitoring of dab is now performed regularly and has excelled as a sentinel for assessing localized contamination and effects on individual fish (Lyons et al. 2006; Stentiford et al. 2009; Vethaak et al. 2009). Temporally stable heterogeneity in the prevalence and aetiology of putatively contaminant-induced biomarkers has been identified around UK coastal waters. For example, the long-term prevalence of benign liver neoplasms ranges from 0 to 24% among localities (Stentiford et al. 2009, 2010), although dab from the North and Irish Seas appear to have a two-year discrepancy between the earliest onsets of the carcinogenic process in each site (Stentiford et al. 2010). Proteomic analysis of dab blood plasma not only reveals their liver tumour status, but also their provenance from either the Irish or North Seas (Ward et al. 2006). Larger sex biases have been reported in expression of hepatic ethoxyresorufin-O-deethylase (EROD) in North Sea fish than in Irish Sea fish (Cefas 2003). The prevalence of hyperpigmentation has been steadily increasing in the North Sea since the 1980s, but not in other areas (Grütjen et al. 2013). Such heterogeneity in disease incidence has been used to classify sites from pristine (Type A, i.e. English Channel and Red Wharf Bay) to relatively impacted (Type C, i.e. Dogger Bank) and is employed to evaluate the success of environmental management and definition of priority areas for restoration or conservation (Stentiford et al. 2009). However, dab have a wide distribution range, from the Bay of Biscay to the White Sea, and there is considerable variation in other phenotypic traits too, such as morphology (e.g. number of vertebrae, spines and rays) and life-history traits (e.g. growth rate, maximum size and age at maturity) (Bakhsh 1982; Deniel 1990; Rijnsdorp et al. 1992a; Henderson 1998). The extent to which such phenotypic and biomarker prevalence heterogeneity is a product of phenotypic plasticity or genotypic variation remains unclear. If variance in phenotypic traits is associated with genetic structuring, then the possibility that variance in biomarker prevalence is due to differential contaminant response by locally adapted dab populations arises (Bickham et al. 2000; Belfiore and Anderson 2001). Variation in biomarker prevalence can be due to various nonmutually exclusive factors: (i) heterogeneity in the distribution of contaminants among sampling sites, (ii) heterogeneity in the age structure among sampling sites resulting in varying duration of exposure, (iii) differential migration dependent on disease status, (iv) different vulnerability to stressors among sampling sites. Disentangling the putative effects of direct exposure to contaminants from age- and population-specific sensitivity and potential migratory patterns therefore becomes of paramount importance for correct interpretations of biomonitoring data and subsequent development of management and/or policies (Theodorakis 2001).

The paradigm of population genetic structure in marine fish has shifted in recent years from the high gene flow and low differentiation model (Hauser and Carvalho 2008), thereby challenging the biomonitoring assumption of uniform response of marine fish to environmental stress. Even with high dispersal potential (Ward et al. 1994; Waples 1998), marked levels of differentiation at both neutral and adaptive markers are now frequently reported, indicating that the scale of differentiation and speed of adaption in marine fish may be at smaller spatial scales and over shorter time intervals than previously predicted (Hemmer-Hansen et al. 2007a, 2013; Larsen et al. 2007, 2012; Williams and Oleksiak 2008; Limborg et al. 2012). Two sampling aspects are decisive in correctly evaluating genetic population structure: temporal replication of the patterns found and targeting reproductively active individuals. Male dab first reproduce at 1 year of age, while females only mature after 2 or 3 years (Bakhsh 1982; Rijnsdorp et al. 1992a). Dab may live up to 11 years (Henderson 1998). The onset of reproductive activity for dab starts in January and ends in September and is characterized by being particularly extended at most locations (van der Land 1991) with individual females spawning for a maximum of 6 weeks (Htun-Han 1978). Eggs undergo passive dispersion and are most abundant at the German Bight, the north of the Friesian Islands, the southern edge of the Dogger Bank, and off Flamborough Head in the North Sea (Rijnsdorp et al. 1992a; Bolle et al. 1994); and all across the Irish Sea but with higher concentrations off Ireland and in Liverpool and Cardigan Bays (Fox et al. 1997). Hatching time is dependent on environmental temperature and ranges from 33 days at 2°C to 4.5 days at 14.5°C (Henderson 1998). In contrast to other flatfish species, dab larvae settlement is not restricted to shallow waters or estuaries (Bolle et al. 1994; Henderson 1998).

As part of the CSEMP, we describe here baseline genetic data on the levels, patterns and stability of genetic structure and diversity in dab around the UK using a panel of species-specific microsatellites (Tysklind et al. 2009). We then evaluate the relative importance of observed population genetic structure, considered in relation to the potential for local adaptation (Carvalho 1993; Conover et al. 2006), and compare it to that of other relevant variables (contaminant exposure, sex, age) in explaining the patterns of biomarker profiles. Potential implications for biomonitoring programmes are then considered.

## Materials and methods

### Sampling

**Figure 1:**
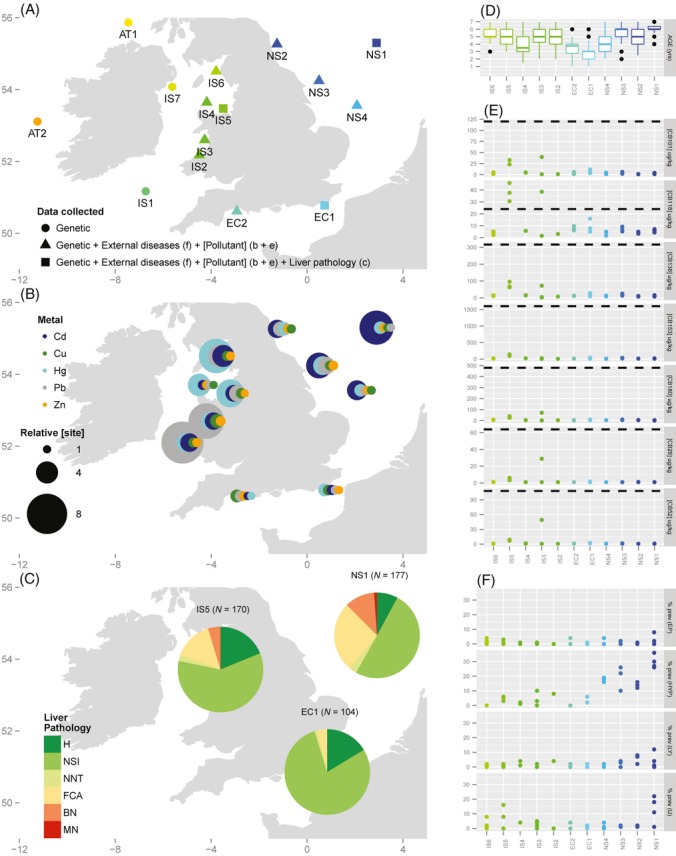
Sampling sites and background data: (A) Dab sampling sites around the British Isles. NS: North Sea sites; EC: English Channel sites; IS: Irish Sea sites; AT: Atlantic sites; Circles: sites where only genetic data are available; Triangles: sites where genetic, external diseases (F) and contaminant exposure (B + E) data are available; Squares: as triangles plus liver pathology (C). Sites are colour-coded to reference other figures (D, E, F, Fig. 2 and Fig. 3). (B) Relative site average dab liver concentration of heavy metals as a multiple of the smallest concentration found in this study [i.e. (Cd) at NS1 is 6.6x(Cd) at EC2]. Cd: cadmium; Cu: copper; Hg; mercury; Pb: lead; Zn: zinc. (C) Prevalence of liver pathologies by category at three sites (NS1, EC1 and IS5) in 2007. N: number of individuals analysed. H: Healthy; NSI: nonspecific inflammatory; NNT: non-neoplastic toxicopathic; FCA: foci of cellular alteration; BN: benign neoplasm; MN: malignant neoplasm. (D) Dab age distribution in years per site (colour-coded, A). (E) Average (*N* = 5) dab liver concentration of 7 chlorinated biphenyls: CB101, CB118, CB138, CB153, CB180, CB28 and CB52 (μg/kg) for up to 4 years per site (colour-coded, A). Environmental quality standards are indicated with a dashed line (OSPAR 2009). (F) Prevalence (%) of external diseases in dab (*N* = 100) for up to 4 years per site (colour-coded, A). EP: epidermal papilloma; HYP: skin hyperpigmentation; LY: lymphocystis; and U: skin ulcers.

Dab have been monitored annually around the UK since the mid-1980s (MAFF 1995) to assess the presence of parasites, diseases and other biomarkers of contaminant exposure (Stentiford et al. 2009). Genetic samples were collected for four consecutive years (2005–2008) in June and July as part of the CSEMP programme in up to 11 sampling stations around the UK. Individuals ranged from fish in spawning condition to recently spent, indicating the sampling of actively breeding individuals. Additionally, four samples were included in 2006 to expand the spatial coverage of genetic analyses (ICES areas: IVb, IVc, VIId, VIIe, VIIa, VIIj, VIa, VIIb) (Fig. 1A). Overall, 39 samples, each with 30 – 183 individuals (with a total of 3006 individuals), were genotyped (Table 1). Samples were allocated a two-letter code and a number representing sea of collection and sampling site (i.e. NS1, EC2, IS4) and two numbers indicating the year of collection (05–08). DNA was extracted from the fin clips using the hi-salt extraction method (Aljanabi and Martinez 1997), and samples were genotyped for 16 microsatellite loci (Tysklind et al. 2009) in multiplex PCR using an ABI 3130xl Genetic Analyzer (Applied Biosystems, Foster City, CA, USA). Allele sizes were determined using GENE MAPPER® Software 4.0 (Applied Biosystems).

**Table 1:** Dab genetic samples: sampling site; CSEMP station number, latitude (Lat.), longitude (Long.) and sample size (number of individual fish genotyped within a sample) sorted by collection year

Site	CSEMP	Lat.	Long.	Sample Size			

				2005	2006	2007	2008
NS1	283	55.3000	2.8970	81	92	183	92
NS2	244	55.2669	−1.2543	42	86	47	95
NS3	344	54.2453	0.4985	–	37	48	94
NS4	378	53.5567	2.0820	–	59	48	100
EC1	486	50.7790	0.7305	–	92	178	96
EC2	536	50.6143	–2.9303	46	77	50	50
IS1	*–*	51.3231	−7.4641	–	94	–	–
IS2	654	52.1816	−4.4978	–	99	–	–
IS3	655	52.3000	−4.2725	99	39	49	30
IS4	776	53.7446	−4.1828	–	50	50	–
IS5	715	53.4720	−3.6985	–	47	183	95
IS6	769	54.5118	−3.7938	30	95	48	96
IS7	–	54.0801	−5.6215	–	64	–	–
AT1	–	55.8680	−7.4541	–	96	–	–
AT2	–	51.1135	−11.2476	–	48	–	–
Total				298	1075	884	748

### Genetic data analysis

Observed (*H*_O_) and expected heterozygosity (*H*_S_), locus-specific fixation indexes (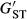) (Nei 1987), and heterozygosity standardized fixation indexes (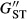) (Meirmans and Hedrick 2011) were all estimated in GENODIVE 2.0b23 (Meirmans and van Tiernderen 2004). Inbreeding coefficients (*F*_IS_), deviations from Hardy–Weinberg equilibrium (HWE) and linkage equilibrium were tested in GENEPOP V4.0 (Rousset 2008) (10 000 dememorizations and 100 batches of 5000 iterations). Given the high number of tests performed, a correction for multiple testing is needed to avoid type I errors. Although Bonferroni correction is commonly used, it can be too stringent and results in type II errors, and hence the sequential goodness-of-fit (SGoF) method (Carvajal-Rodríguez et al. 2009), which is particularly suited to high-dimensional data, was used to correct for multiple testing for all genetic data. The presence of null alleles, large allele dropout and scoring errors due to allele stuttering were assessed with MICRO-CHECKER (van Oosterhout et al. 2004). Loci with suspected null alleles were excluded from analyses except where otherwise stated.

### Population structure

Several complementary methods were used to investigate population structure among samples. Exact *G*-tests of the probability of random allele distributions across samples were calculated using GENEPOP V4.0 (10 000 dememorizations and 500 batches of 10 000 iterations). Global and pairwise 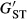 and heterozygosity standardized 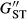, and associated statistical difference from zero based on 9999 permutations were calculated using GENODIVE 2.0b23. Each year was treated independently. Each sample does not necessarily represent a unique population, as several samples may be collected from the same population and samples may contain individuals from several populations. The number of populations was estimated by the K-means clustering analysis available in GENODIVE 2.0b23. Analysis of molecular variance (amova) (Excoffier et al. 1992) estimating *F*-statistics and their heterozygosity standardized versions (Meirmans 2006) were performed in GENODIVE 2.0b23 using 999 permutations and assuming infinite alleles. Variation within and among individuals, samples and clusters (as estimated by the K-means clustering) was estimated for each year separately. Sample correspondence analysis (CA) of the whole data set was performed using the R package, ADEGENET (Jombart 2008). As recommended by the author, CA plots were centred on the origin and missing genotypes (1.65% for 16 loci) replaced with the mean χ^2^ distance, which effectively places missing data at the origin of the axes.

### Temporal analysis

Several methods were employed to assess the stability of genetic structure over the temporal replicate samples. First, pairwise 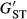 and 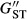 were calculated for samples across years within site, and correlation between 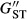 and time (years) was studied with Mantel tests in GENODIVE 2.0b23. Secondly, to evaluate stability of general genetic distance among samples across years, the correlation between genetic distances (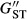) among years across the whole study area was also tested using Mantel tests.

### Age and sex effects on genetic differentiation

Cohort strength fluctuations have been reported for dab (Henderson 1998), which could generate apparent genetic structuring (Mariani et al. 2005). Age data, obtained from otoliths, were supplied by the Centre for Environment, Fisheries and Aquaculture Science (Cefas) for three samples in 2007 (NS1-07, EC1-07 and IS5-07) with 178–184 individuals each. To evaluate whether there were cohort changes in genetic composition within sites, the samples were subdivided into age classes and an analysis of genetic differentiation among age classes within site was performed for all subclasses with at least 20 individuals (from age 1 up to age 5). Male and female dab are known to have independent migration patterns (Saborowski and Buchholz 1997), and hence, the effect of sexes was evaluated in GENODIVE 2.0b23 by: first, amovas to test whether both sexes were genetically structured across the sampling range (variance due to samples and clusters within sexes); second, Mantel tests of correlation between pairwise 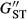 among males and females; third, amovas to test whether sexes were structured within samples (variance due to sexes within samples); and finally, the OSx-statistic (Goudet 1995) to test whether males and females differed in G-statistics (*H*_O_, *H*_S_, *G*_IS_, 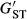, and 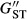).

### Assembling of biomarker, contaminant exposure, age, sex and genetic data

All contaminant and disease data were collected on behalf of the Clean Safe Seas Evidence Group and obtained via the Marine Environment Monitoring and Assessment National database (MERMAN) (http://www.bodc.ac.uk/projects/uk/merman/). Two levels of biomarkers are considered here, each composed of several indicators and collectively encompassing a range of sensitivities to contaminant exposure

*External prevalence of four grossly visible diseases*: lymphocystis (LY), epidermal papilloma (EP), skin ulceration (U) and skin hyperpigmentation (HYP), among 100 individuals were recorded in each of 35 samples (Fig. 1F), providing a spatial (up to 11 sites) and temporal (up to 4 years) insight into the aetiology of the diseases at the sample level.

*Liver pathology at the individual level*: prevalence of 32 different liver lesions was classified into five categories (Feist et al. 2004): nonspecific inflammatory (NSI), non-neoplastic toxicopathic (NNT), foci of cellular alteration (FCA), benign neoplasm (BN) and malignant neoplasm (MN) (Fig. 1C). Fish with none of these lesions were considered healthy (H). Data were available at the individual level for the three age-classified samples in 2007 (NS1-07, EC1-07, IS5-07, *N* = 451), providing a more detailed examination of age, population and exposure on liver lesions, particularly neoplastic processes.

Sex and age were available for individuals in the liver pathology exercise, while site age averages were obtained from the 2004 CSEMP assessment for the external analysis (Fig. 1D). We assume that mean age is a relatively stable characteristic of site, because biomarker prevalence is stable over time (Stentiford et al. 2009) and biomarkers are also a good indicator of age (Stentiford et al. 2010). Age averages from 2007 are congruent with those from 2004. Sex was not included as a factor in the external analysis as it was not recorded for all samples.

Estimates of contaminant exposure were obtained from the CSEMP data. Contaminant exposure is evaluated every year for five individuals per station (five individuals per age class in the age-classified samples). Heavy metal (Cd, Cu, Hg, Pb, Zn) and seven polychlorinated biphenyls (CB101, CB118, CB138, CB153, CB180, CB28, CB52), were measured from liver tissue. Averages of these values (within site and year) were used as site × year exposure estimates (Fig. 1B and Fig. 1E). With the exception of CB118 in IS3 and IS5, all other PCBs are below environmental quality standards (Fig. 1E). Copper and zinc concentrations were not available for all years and hence were not included in the external assessment. Polychlorinated biphenyls were not available for all age classes and hence were not included in the liver pathology assessment.

As the correspondence analysis summarizes the complexity of the microsatellite data, the values along the axes were used as proxies of the effect of population genetic structure. To evaluate any relationship between biomarkers and genetic diversity or erosion, multilocus *H*_O_ and *F*_IS_ values were included as variables at the population level. At individual level, two measures of individual inbreeding were calculated: internal relatedness (*IR*) (Amos et al. 2001), based on the allele frequency–corrected ‘relatedness’ of the two alleles at each locus averaged over loci, and homozygosity weighted by loci, *hL* (Aparicio et al. 2006), which takes locus allelic diversity into consideration. Both were estimated in STORM v1.1 (Frasier 2008).

### Evaluations of variable importance in biomarker prevalence

Although disease prevalence in dab is spatially heterogeneous among target sites, such patterns are temporally stable (Stentiford et al. 2009). In biomonitoring exercises, biomarker prevalence is assumed to be representative of contaminant exposure. However, other variables such as age have been found to play an important role in disease prevalence in dab (Stentiford et al. 2010), and variance in cancer prevalence has been found among human populations (Kiyohara et al. 2005; Carey et al. 2006; Distelman-Menachem et al. 2009). Here, we aim to evaluate the relative importance of population membership (defined genetically), age and contaminant exposure in determining biomarker prevalence in dab around the UK through the construction of classification trees and *random forest* methods (Breiman 2001). Such methods are appropriate for data where nonlinear relationships between predictive and response variables and complex interactions between variables, such as covariance, may be expected (Cutler et al. 2007; Strobl et al. 2007). Classification trees allow the visualization of models predicting response based on several variables. Recursive binary classification of the data is achieved through, first, testing for independence between the covariates (here: age, contaminants and genetics) and the response (biomarker prevalence); second, if dependence is found, determining the best split value for the covariate with the strongest effect on the response; and third, repetition of the first two steps with each of the branches until independence cannot be rejected (Hothorn et al. 2006). Random forest improves the prediction accuracy by first generating bootstrap subsamples of the original data with a reduced number of predictor variables and then growing unpruned binary classification trees for each subsample. Prediction is then based on majority vote from the whole forest (Breiman 2001). Random forest can also be used to assess variable importance, where variables are randomly permuted to evaluate which variables ensue in an increase in prediction error when removed (Strobl et al. 2007). Variable importance was evaluated in the R package, PARTY (Hothorn et al. 2006) with the cforest_unbiased function, which avoids bias introduced by heterogeneity in scale and number of categories among variables (Strobl et al. 2007). Variable importance scores depend on the scales and number of categories of variable measurement, and hence, absolute values are normally not interpreted (Strobl et al. 2008). To remove the effect of variance in scale and allow a more intuitive comparison among responses, a second evaluation was performed where variables were standardized before analysis. The number of trees was set to 5000 to avoid random variation in importance ranking (Strobl et al. 2009). Conditional inference trees were also constructed in PARTY, with 10 000 resamples of Monte Carlo–adjusted *α* = 0.05 significance levels and with at least two observations into each daughter node.

## Results

### Microsatellite locus characteristics and conformity to expectations

The number of alleles per locus ranged from 8 to 56, and the observed heterozygosity per locus was between 0.089 and 0.946 (Table S1). The mean observed heterozygosity across loci within samples was between 0.666 and 0.728. Two markers, *DAC1-35* (*F*_IS_ = 0.139) and *DAC5-70* (*F*_IS_ = 0.371), showed heterozygote deficiencies (Table S1). MICRO-CHECKER suggested the presence of null alleles in some of the samples in some of the years for *DAC1-35* and for all samples in all years for *DAC5-70*. No sample–locus combination showed evidence of stuttering errors or large allele dropout. Once the two markers deviating from HWE were removed, all samples complied with HWE expectations (all multilocus *P*-values >0.05, Table S2) and no single locus–sample combination was significant after multiple testing correction. No evidence of linkage disequilibrium was found among any combination of loci in any of the samples once corrected for multiple testing.

### Levels of genetic structuring among samples and pairwise comparisons

**Figure 2:**
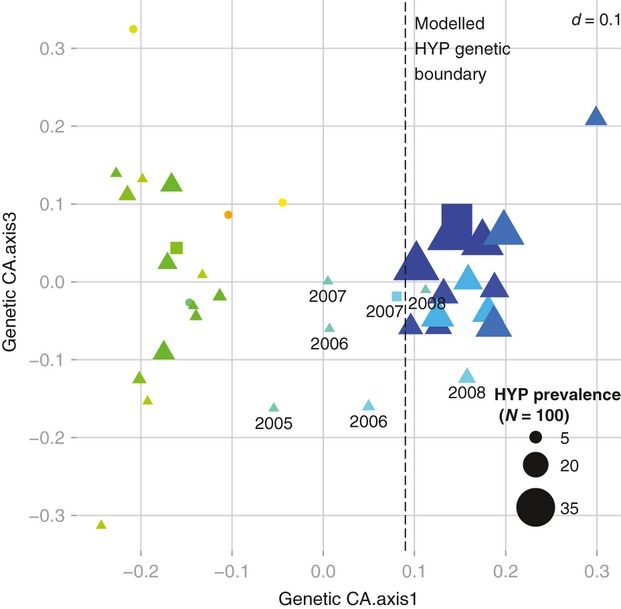
Sample correspondence analysis and HYP prevalence. Sample genetic correspondence analysis (CA) of dab samples (colour-coded, Fig. 1A) separating North Sea (dark blues) and English Channel (cyan) from Irish Sea (greens) and Atlantic samples (oranges) along the first axis (CA.axis1). The prevalence of hyperpigmentation (HYP; *N* = 100) is overlaid on top of the genetic data and represented by size. The genetic boundary identified through the disease modelling is indicated through a discontinuous line (HYP: dashed). Collection years for the English Channel samples are indicated below the sample.

**Figure 3:**
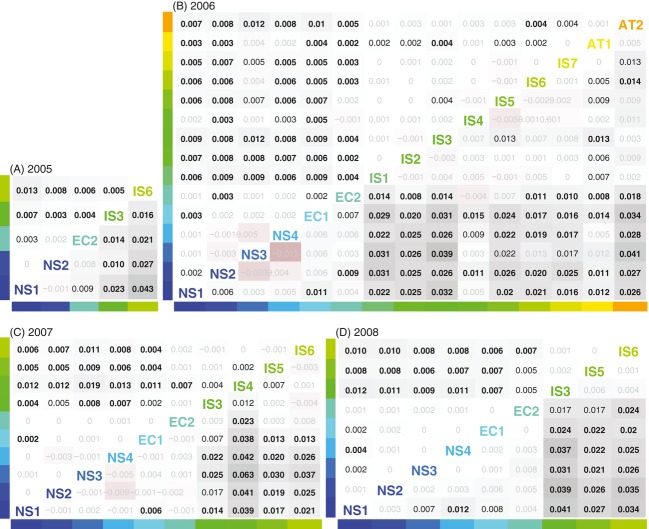
Pairwise estimates of differentiation (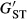 *&* 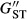) of dab around the British Isles. Pairwise estimators of differentiation between dab samples (colour-coded, Fig. 1A), sorted by years: (A) 2005; (B) 2006; (C)2007; (D)2008. Tables correspond to pairs of 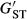 (above diagonal) and heterozygosity standardized 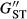 (below diagonal). Differentiation values are represented as grey heatmaps: the darker the grey, the higher the relative value among comparisons (within year). Differentiation values significantly different from zero are in black; those still significant after correction for multiple testing are in boldface.

The global 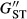 = 0.015 (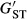 = 0.004; *P* < 0.001), which measures the level of population structuring, including all samples and markers, suggested weak structuring of dab samples. There were, however, marked and consistent differences in allele frequencies for some loci among sites. The patterns of genic subdivision indicated that the allele distribution was consistently significantly different from random (*P* < 0.05) for several loci (38 of 64 loci–year comparisons), further suggesting the existence of genetic structure. Such allelic structure is illustrated in the CA (Fig. 2), which separated all North Sea samples from Irish Sea and Atlantic samples along the first axis, placing the EC2 samples of 2005, 2006 and 2007 in between North Sea and Irish Sea samples. The EC2-08 sample clustered with the rest of the North Sea samples. Pairwise estimates of population differentiation (Fig. 3) ranged from negative values (=0, indicating no structure) to a maximum 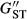 of 0.063 (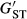 of 0.019). With the exception of a few values discussed later, differentiation estimates were close to zero and nonsignificant between samples from the same sea basin (i.e. North Sea, Irish Sea), but higher and highly significant (*P* < 0.001) when comparing samples across sea basins. The pattern between North Sea and Irish Sea was consistent across years. Within the North Sea, NS1 samples were significantly differentiated from some of the near-shore North Sea and English Channel samples in some of the years (Fig. 3). Dab from the Irish Sea were also genetically homogeneous with very few significant genetic differences among samples, which furthermore were not repeated over time (Fig. 3). The English Channel samples displayed varying levels of structure in different years. The two Atlantic samples from 2006 displayed highly significant (*P* < 0.001) genetic differentiation when compared to most North Sea samples and some Irish Sea samples. The K-means clustering analysis suggested that *k* = 2 best fitted the data for all years, separating all North Sea and English Channel samples in one cluster from those collected in the Irish Sea and Atlantic. The amova (Table 2) suggested that approximately 98.4% of the variation was found within individuals, while 0.9, 0.1 and 0.5% were among individuals within samples, among samples within clusters and among clusters, respectively. Despite low levels, all values were significant (all *P* < 0.001). When *F*-values were corrected for high heterozygosity, differentiation among samples within clusters remained low (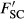 = 0.4%), but became more evident between clusters (up to 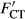 =2.4% in 2008).

**Table 2:** amova of dab around the British Isles over four years (2005–2008)

					Whole					Females					Males		
	Source of Variation	Nested in	%var	*F*-value	st.err.	*P*-value	*F*′-value	%var	*F*-value	st.err.	*P*-value	*F*′-value	%var	*F*-value	st.err.	*P*-value	*F*′-value
2005	Within Individual	–	0.981	0.019	0.011	–	–	0.977	0.023	0.014	–	–	0.979	0.021	0.007	–	–
	Among Individual	Samples	0.012	0.012	0.008	0.040	–	0.017	0.017	0.012	0.032	–	0.015	0.016	0.007	0.036	–
	Among Samples	Clusters	0.003	0.003	0.002	**0.006**	0.010	0.006	0.006	0.003	**0.006**	0.019	0.003	0.003	0.002	0.041	0.011
	Among Clusters	–	0.004	0.004	0.002	**<0.001**	0.013	0.001	0.001	0.002	0.114	0.002	0.003	0.003	0.002	**<0.001**	0.009
2006	Within Individual	–	0.986	0.014	0.008	–	–	0.991	0.009	0.010	–	–	0.983	0.017	0.010	–	–
	Among Individual	Samples	0.008	0.008	0.006	**0.013**	–	0.004	0.004	0.007	0.224	–	0.008	0.009	0.009	0.054	–
	Among Samples	Clusters	0.001	0.001	0.000	**<0.001**	0.005	0.002	0.002	0.001	0.015	0.006	0.002	0.002	0.001	**0.017**	0.006
	Among Clusters	–	0.004	0.004	0.003	**<0.001**	0.014	0.003	0.003	0.002	**<0.001**	0.012	0.007	0.007	0.004	**<0.001**	0.022
2007	Within Individual	–	0.985	0.015	0.010	–	–	0.980	0.020	0.011	–	–	0.997	0.003	0.013	–	–
	Among Individual	Samples	0.009	0.009	0.008	**0.008**	–	0.015	0.015	0.008	**<0.001**	–	−0.004	−0.004	0.011	0.705	–
	Among Samples	Clusters	0.000	0.001	0.001	0.088	0.002	0.001	0.001	0.001	0.081	0.003	0.000	0.000	0.002	0.414	0.001
	Among Clusters	–	0.005	0.005	0.003	**0.005**	0.018	0.005	0.005	0.003	**<0.001**	0.016	0.007	0.007	0.004	**<0.001**	0.022
2008	Within Individual	–	0.984	0.016	0.008	–	–	0.981	0.019	0.009	–	–	0.987	0.013	0.010	–	–
	Among Individual	Samples	0.008	0.008	0.007	0.036	–	0.009	0.009	0.007	**<0.001**	–	0.005	0.005	0.010	0.241	–
	Among Samples	Clusters	0.001	0.001	0.001	**0.009**	0.004	0.001	0.001	0.001	0.160	0.003	0.002	0.003	0.001	**0.020**	0.009
	Among Clusters	–	0.007	0.007	0.003	**<0.001**	0.024	0.009	0.009	0.004	**0.012**	0.030	0.005	0.005	0.002	**0.013**	0.018
All years	Within Individual	–	0.985	0.015	0.009	–	–	0.981	0.019	0.010	–	–	0.986	0.014	0.010	–	–
	Among Individual	Samples	0.009	0.009	0.006	**<0.001**	–	0.013	0.013	0.007	**<0.001**	–	0.007	0.007	0.007	0.026	–
	Among Samples	Clusters	0.001	0.001	0.000	**<0.001**	0.004	0.001	0.001	0.000	**<0.001**	0.004	0.001	0.001	0.001	**0.006**	0.003
	Among Clusters	–	0.005	0.005	0.003	**<0.001**	0.017	0.005	0.005	0.003	**<0.001**	0.018	0.007	0.007	0.004	**<0.001**	0.022

			Between sexes for HWE loci					Between sexes without *DAC4-40*					Between sexes: *DAC4-40*					
	Source of Variation	Nested in	%var	*F*-value	st.err.	*P*-value	*F*′-value	%var	*F*-value	st.err.	*P*-value	*F*′-value	%var	*F*-value	st.err.	*P*-value	*F*′-value
2005	Within Individual	–	0.979	0.021	0.008	–	–	0.977	0.023	0.009	–	–	0.976	0.024		–	–
	Among Individual	Sexes	0.015	0.015	0.007	**0.009**		0.018	0.018	0.008	**0.003**		0.002	0.003		0.485	–
	Between Sexes	Samples	0.003	0.003	0.002	**0.014**	0.010	0.002	0.002	0.001	0.072	0.006	0.016	0.016		**<0.001**	0.121
	Among Samples	–	0.003	0.003	0.002	**<0.001**	0.011	0.003	0.003	0.002	**<0.001**	0.010	0.005	0.005		**<0.001**	0.043
2006	Within Individual	–	0.987	0.013	0.008	–	–	0.985	0.015	0.008	–	–	1.005	−0.005		–	–
	Among Individual	Sexes	0.009	0.009	0.006	**0.008**	–	0.012	0.012	0.007	**0.002**	–	−0.017	−0.017		0.939	–
	Between Sexes	Samples	0.001	0.001	0.001	0.052	0.004	0.000	0.000	0.001	0.645	−0.001	0.015	0.015		**<0.001**	0.116
	Among Samples	–	0.003	0.003	0.002	**<0.001**	0.011	0.004	0.004	0.002	**<0.001**	0.012	−0.003	−0.003		**<0.001**	−0.022
2007	Within Individual	–	0.985	0.015	0.010	–	–	0.983	0.017	0.011	–	–	0.999	0.001		–	–
	Among Individual	Sexes	0.011	0.011	0.008	**0.002**	–	0.013	0.013	0.009	**<0.001**	–	−0.014	−0.015		0.902	–
	Between Sexes	Samples	0.003	0.003	0.003	**<0.001**	0.009	0.000	0.000	0.000	0.594	−0.001	0.030	0.030		**<0.001**	0.227
	Among Samples	–	0.002	0.002	0.003	**<0.001**	0.007	0.004	0.004	0.002	**<0.001**	0.012	−0.014	−0.014		**<0.001**	−0.137
2008	Within Individual	–	0.981	0.019	0.007	–	–	0.977	0.023	0.008	–	–	0.973	0.027		–	–
	Among Individual	Sexes	0.015	0.015	0.007	**<0.001**	–	0.016	0.016	0.006	**<0.001**	–	0.022	0.023		0.709	–
	Between Sexes	Samples	0.001	0.001	0.001	0.072	0.003	0.001	0.001	0.001	0.224	0.002	0.003	0.003		**0.004**	0.066
	Among Samples	–	0.004	0.004	0.001	**<0.001**	0.013	0.006	0.006	0.003	**<0.001**	0.021	0.001	0.001		**<0.001**	−0.018
All years	Within Individual	–	0.985	0.015	0.007	–	–	0.984	0.016	0.008	–	–	0.999	0.001		–	–
	Among Individual	Sexes	0.010	0.010	0.006	**<0.001**	–	0.012	0.012	0.006	**<0.001**	–	−0.010	−0.010		0.942	–
	Between Sexes	Samples	0.002	0.002	0.002	**<0.001**	0.006	0.000	0.000	0.000	0.244	0.001	0.018	0.017		**<0.001**	0.140
	Among Samples	–	0.003	0.003	0.002	**<0.001**	0.009	0.004	0.004	0.002	**<0.001**	0.012	−0.006	−0.006		**<0.001**	−0.057

Sources of variation: individuals; individuals within samples; samples within clusters; and among clusters. Clusters are those estimated by the K-means clustering analysis and correspond to North Sea–English Channel and Irish Sea–Atlantic basins. %var = percentage of variation explained by the source of variation. *F*-value = estimated amount of differentiation in the system due to each source of variation. St.err = standard error around the estimated *F*-value. *P*-value = probability that the 95% confidence interval of the estimated *F*-value encompasses zero. *F*′-value: estimated amount of differentiation in the system after standardization for heterozygosity. The analysis was repeated with only females and only males. The variation among sexes within samples was evaluated for all HWE loci, for all HWE except locus *DAC4-40* and for locus *DAC4-40* alone.

Boldface values indicate significant probability values after multiple testing correction (Carvajal-Rodríguez et al. 2009) within each test (i.e. whole, females…).

### Temporal analysis

All pairwise 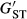 and 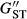 values for samples from the same site across years were low (average 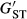
0.001, average 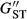 = 0.004), and none remained significant after multiple testing correction (Carvajal-Rodríguez et al. 2009), indicating temporal stability at sampling sites. Except for the comparison between 2005 and 2006 (*r* = 0.303, *P* = 0.157), the patterns of genetic distances (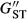) among samples each year were significantly correlated (*P* < 0.05) with each other (Table 3), indicating that the relative genetic differentiation among samples was also temporally stable. Despite the general within-site genetic stability, a noteworthy trend was found in the western English Channel: EC2 samples became increasingly more differentiated over time, a relationship revealed by a Mantel test (*r* = 0.922; *P* < 0.001) and also displayed by the CA (Fig. 2), initially locating them close to Irish Sea samples in 2005, then displacing them towards the North Sea in 2006 and 2007 and finally placing them amidst all North Sea samples in 2008.

**Table 3:** Mantel test of correlation of genetic distances (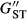) of dab samples among years, and between sexes across the whole study area for each of the years

	2006		2007		2008			Between sexes	
					
	*r*	*P*-value	*r*	*P*-value	*r*	*P*-value		*r*	*P*-value
2005	0.303	0.157	0.622	**0.015**	0.496	**0.034**	2005	−0.019	0.500
2006			0.382	**0.026**	0.862	**0.003**	2006	0.360	**0.007**
2007					0.821	**0.002**	2007	0.401	**0.014**
							2008	0.056	0.423
							2008*	0.561	0.050

Significant probability values (*P*) after multiple testing correction are in boldface.

* 2008*: correlation values for 2008 samples after removal of samples with less than 10 individuals in either sex..

### Age and Sex effects on genetic differentiation


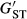
 values between age classes within samples were very low (negative to 0.001) and nonsignificant, suggesting genetic homogeneity among year cohorts. In those samples for which age and sex data were available, the proportion of females increased with age, as reported elsewhere (Deniel 1990). Both sexes of dab when analysed separately were structured across the sampling range (amova, Table 2) and pairwise 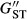 estimates among males and females were significantly correlated for 2006 and 2007, but not in 2005 and 2008. Lack of correlation in some years is probably due to unequal partitioning of males and females among samples, as small subsamples sometimes resulted in high, albeit not significant, 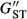. None of the G-statistics compared between males and females were significantly different from each other after multiple testing corrections (Table 4). Altogether, we found no evidence of sex-specific demographic patterns affecting the distribution of neutral genetic diversity. However, amovas evaluating the variance due to sexes within sample were significant for some of the years (2005, 2007). Detail examination of amovas by loci revealed that one of the markers (*DAC4-40*) was responsible for most of such variance among sexes within samples, as amovas became nonsignificant after the removal of *DAC4-40* (Table 2). Despite being in HWE (*P* = 0.312 across all samples), the very high among-sexes *F′-*values (up to 0.227; Table 2) are due to differences in *H*_O_ among sexes, where males show heterozygosity excess (Table 4). Removal of *DAC4-40* had no impact in the distribution of samples in the CA or significances of sample differentiation values (data not shown).

**Table 4:** Test of significant difference in G-statistics between sexes of dab across years for all HWE loci and for locus *DAC4-40* alone for all years combined

Year/Locus	Statistic	Females	Males	OSx	*P*-value
2005	Ho	0.672	0.699	0.026	0.103
	Hs	0.685	0.711	0.026	0.025
	Gis	0.019	0.017	0.001	0.851
	G′st	0.010	0.005	0.005	0.236
	G″st	0.032	0.018	0.014	0.236
2006	Ho	0.698	0.698	0.000	0.946
	Hs	0.705	0.706	0.001	0.812
	Gis	0.009	0.011	0.002	0.839
	G′st	0.004	0.005	0.001	0.477
	G″st	0.013	0.017	0.004	0.473
2007	Ho	0.695	0.697	0.002	0.762
	Hs	0.705	0.700	0.005	0.374
	Gis	0.014	0.004	0.010	0.324
	G′st	0.004	0.004	0.000	0.980
	G″st	0.015	0.015	0.000	0.993
2008	Ho	0.698	0.696	0.003	0.822
	Hs	0.705	0.711	0.006	0.478
	Gis	0.009	0.022	0.013	0.439
	G′st	0.000	0.006	0.005	0.335
	G″st	0.001	0.020	0.019	0.331
*DAC4-40 (all years)*	Ho	0.864	0.928	0.064	**<0.001**
	Hs	0.874	0.877	0.002	0.75
	Gis	0.012	−0.059	0.071	**<0.001**
	G′st	0.005	0.01	0.005	0.339
	G″st	0.041	0.08	0.039	0.281

Significant values after multiple testing correction are in boldface.

### Evaluation of variable importance in biomarker prevalence

**Figure 4:**
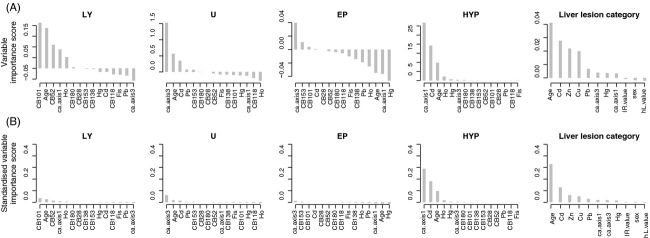
Variable importance scores for biomarker prevalence in dab. (A) Conditional variable importance scores, in rank order, of variables assessed for various dab biomarkers: lymphocystis (LY), skin ulcers (U), epidermal papilloma (EP), skin hyperpigmentation (HYP) and liver lesion category. (B) Conditional variable importance scores of standardized variables.

**Figure 5:**
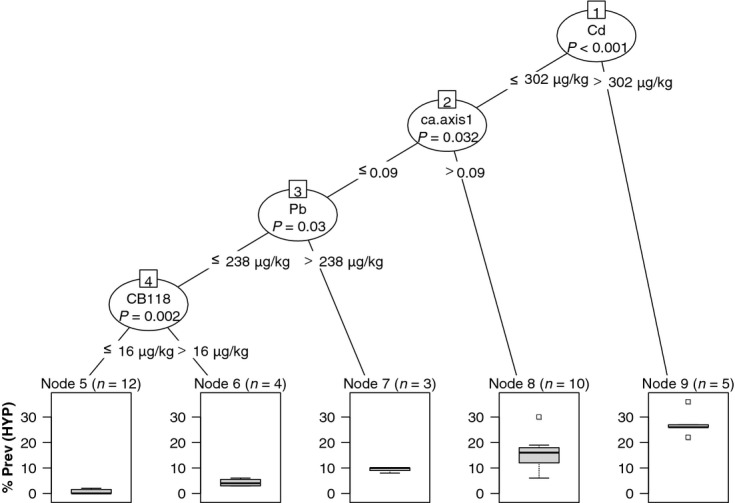
Conditional inference tree of hyperpigmentation (HYP) prevalence in dab. Bubbles indicate the variable with the strongest effect on the response and associated probability (Monte Carlo–adjusted *α* = 0.05). Boxplots in each node summarize the per cent prevalence of HYP among dab in sites classified into each node (n).

Standardizing the data before variable importance analysis did not affect the ranking of the most important variables for each biomarker. However, it allowed distinguishing biomarkers significantly correlated with variables, as evaluated by the conditional inference trees, from those where no significant dependencies between any of the variables and biomarkers could be found. Three of the biomarkers, LY, U and EP, had low standardized scores (Fig. 4), and conditional inference trees reported a single final node for all of them, suggesting little explanatory power for the variables assessed here. Conversely, population structure (CA.axis1) and cadmium exposure were good predictors of HYP prevalence (Fig. 4). The conditional inference tree suggested that several variables had a significant effect on HYP prevalence: samples with average cadmium liver concentration above 302 μg/kg had the highest prevalence of HYP (Fig. 5), followed by those samples to the right of CA.axis1 = 0.09, namely North Sea sites (Fig. 2); of the sites in the English Channel and Irish Sea, higher HYP prevalence was associated with high lead exposure (>238 μg/kg). The lowest HYP prevalence was associated with low exposure (<16 μg/kg) to a polychlorinated biphenyl, CB118 (Fig. 5).

**Figure 6:**
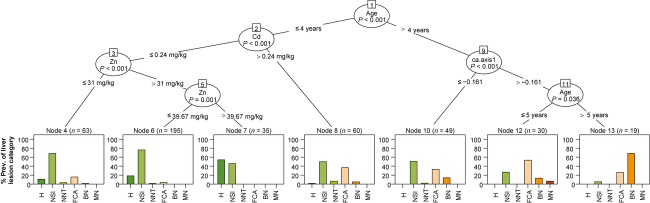
Conditional inference tree of liver lesion category in dab. 32 liver lesions are summarized into: no lesions (H), nonspecific inflammatory (NSI), non-neoplastic toxicopathic (NNT), foci of cellular alteration (FCA), benign neoplasms (BN) and malignant neoplasms (MN). Bubbles indicate the variable with the strongest effect on the response and associated probability (Monte Carlo–adjusted *α* = 0.05). Histograms represent the per cent prevalence of each category among the individuals classified in each node (n).

The conditional inference tree of the individual liver pathology classification highlighted how age, population structure and contaminant exposure all interacted and had significant effects on liver health (Fig. 6). Age was the most important variable (Fig. 4), whereby older fish had increased prevalence of liver pathologies and no fish above 4 years of age were assigned to the ‘healthy’ category. The frequency of the different pathologies among older fish, however, differed among genetic populations: old fish from Liverpool Bay experienced higher nonspecific inflammatory diseases, while old fish from the North Sea exhibited higher prevalence of neoplastic processes. Among North Sea dab, those of 5 years of age seem to be more affected by foci of cellular alteration and malignant neoplasms and fish over 5 years of age had a higher frequency of benign neoplasms (Fig. 6). In younger fish, contaminants seem to play an important role in liver pathology classification (regardless of population structure). High exposure to cadmium is associated with early onset of preneoplastic lesions, while high levels of zinc are associated with healthy livers and low prevalence of nonspecific inflammatory diseases. However, it is important to note that liver zinc concentration was negatively correlated with age (Pearson's *r* = −0.460; *P* = 0.038), especially under 4 years of age (Pearson's *r* = −0.732; *P* = 0.006).

None of the measures of genetic diversity at either samples or individual level (*H*_O_, *F*_IS_, *IR* and *hL*) were correlated with any of the biomarkers.

## Discussion

### Dab population structure

Given that dab is the third most common fish in the North Sea (Daan et al. 1990), the relatively recent colonization by North and Irish Sea biota after the last glacial maximum (Maggs et al. 2008), and potential for egg and larval dispersal (Henderson 1998), little genetic structuring may be expected. Contrary to such expectations, temporally stable patterns of significant differentiation were observed. Dab around the British Isles exhibit detectable genetic structure: individuals from the North Sea were consistently genetically distinct from those in the Irish Sea as revealed by classical (Fig. 3), multivariate (Fig. 2) and Bayesian methods (Tysklind 2009). Genetic structuring within basins, while sometimes significant, was more subtle. Once basins were taken into account, significant isolation-by-distance patterns disappeared (Tysklind 2009), suggesting that the distribution of genetic diversity in dab was more likely influenced by coastal, oceanographic and/or biological features than geographical distance *per se*. Although many studies have evaluated population structure of European flatfish in the North East Atlantic (Danancher and Garcia-Vazquez 2011), relatively few have included samples from both the Irish and North Sea. Among them, very subtle differentiation between North and Irish Seas has been detected in sole, *Solea solea* (Cuveliers et al. 2012), but not in other closely related species such as flounder, *Platichthys flesus* (Galleguillos and Ward 1982; Hemmer-Hansen et al. 2007a,b), and plaice, *Pleuronectes platessa* (Hoarau et al. 2002, 2004, 2005; Was et al. 2010). Compared to other flatfish, dab appear to be more genetically structured across the range studied here. Nevertheless, comparison among studies is difficult given the diversity of markers, spatial and temporal sampling intensity and analytical methods employed.

Several nonmutually exclusive hypotheses may lead to detectable structure in the marine environment: fidelity to spawning site (Thorrold et al. 2001), reduced migration combined with genetic drift (Borsa et al. 1997), and local adaptation (Carvalho 1993; Conover et al. 2006; Hemmer-Hansen et al. 2007a). The existence of genetic structure at a regional level in dab may thus be explained by a combination of dab biology and oceanographic currents. Flatfish have in general been shown to exhibit homing behaviour (de Veen 1978; Rijnsdorp et al. 1992b; Hunter et al. 2003), and dab has been shown to undergo within-basin migrations and return to capture location after a year (Rijnsdorp et al. 1992a), which is compatible with patterns of genetic structuring found here. Dab eggs and larvae are ubiquitous throughout the North (Rijnsdorp et al. 1992a) and the Irish Seas (Fox et al. 1997). Eggs can, however, hatch quickly (4.5 days at 14°C), and larvae seem capable of controlling, to some extent, their movements (Henderson 1998), which together with their ability to settle both inshore or offshore waters (Bolle et al. 1994) may reduce the dispersal of most individual eggs. By studying seven different species, Galarza et al. (2009) showed that marine currents and sea fronts can represent effective barriers to gene flow between populations, regardless of egg type or pelagic larval duration, resulting in detectable genetic differentiation at regional scales. The cyclonic gyre that forms within the Irish Sea in spring and summer has been suggested as a larval retention mechanism for Norway lobster, *Nephrops norvegicus*, (Hill et al. 1996), and could have a similar effect on dab eggs and larvae. Furthermore, a strong jet-like westward flow across the St. Georges Channel (between St. Davids Head of Wales and Carnsore Point in Ireland) prevents the mixing of Celtic and Irish Sea waters (Brown et al. 2003), further constraining the exchange of eggs and larvae between the English Channel and North Sea. An egg transport model incorporating oceanographic data of the Irish Sea (van der Molen et al. 2007), suggested that eggs and larvae dispersed an average of 80 km from point of release and largely remained within 160 km of point of release, with very few dispersing up to 300 km from point of release. Such distances and the oceanographic features of the Irish Sea are in accordance with the genetic structure observed here in dab between North and Irish Seas, for which a major genetic boundary exists between IS3 and EC2 dab, suggestive of few migrants during the early life-history stages of dab.

### Temporal aspects of genetic structure

The stability of genetic structuring over time indicates that it is not the outcome of the random distribution of genetic diversity, but more likely the result of biological processes shaping such distribution and thus strengthening inferences on the biological significance of those differences found (Carvalho and Hauser 1998; Waples 1998; Waples et al. 2008). In dab, the temporal stability of genetic structure across consecutive sampling years was marked, a pattern shared with sole (Cuveliers et al. 2011, 2012), but not plaice (Hoarau et al. 2005). Temporal stability was evident even among age cohorts for those samples for which age information was available (NS1-07, EC1-07 and IS5-07). Such patterns indicate that the stability of genetic composition was inherent to the local dab population, as found in other species (Jorde et al. 2007; Cuveliers et al. 2011), and not a chance effect of repeatedly sampling individuals born in the same year across the four sampling years. Conversely, the instability of the genetic relationships between EC2 and proximate samples is suggestive of an increase in the proportion of North Sea dab into the western English Channel and highlights that the putative boundary between North Sea and Irish Sea dab is probably transient, necessitating regular resampling.

### Relevance of population genetic structure to biomarker prevalence

The significant and temporally stable genetic differences between North and Irish Sea dab indicate that they have the potential to evolve (through drift and adaptation) independently. Under such a scenario, regional differences in dab morphological traits (Bakhsh 1982; Deniel 1990; Rijnsdorp et al. 1992a; Henderson 1998), and most importantly here, the stable variation in different biomarker responses between North and Irish Seas (Cefas 2003; Ward et al. 2006; Stentiford et al. 2009; Tysklind 2009), could be population-specific. It follows that such patterns may represent not only phenotypic plasticity to local conditions (and contaminants), but raises the possibility they may comprise a genetic component.

No measured variables (average age, population genetic structure, cadmium, lead, mercury and 7 PCBs) were associated with prevalence of three of the external biomarkers: LY, EP and U, indicating that either: (i) other contaminants not considered here may play a role in the prevalence of these diseases, (ii) studying their prevalence at sample level was not appropriate, and an individual model may be more adequate, (iii) they are not biomarkers of contaminant exposure. In accordance with the latter, Stentiford et al.(2009) found that LY, EP and U had relatively low discriminatory power at discerning among CSEMP sites. Causality of the fourth external biomarker, HYP, is still debated: no evidence of infectious processes has been found (Noguera et al. 2013), and therefore, other causes such as variance in UV radiation, developmental processes and environmental factors must be explored. Our results indicated significant correlations with several contaminants (Cd, Pb and CB118) and population genetic structure. Such findings allow the definition of population-specific baseline levels of HYP and potentially link changes in HYP prevalence to particular contaminants. For example, among the low-HYP-prevalence Irish Sea population, only sites with high Pb (IS3) and CB118 concentrations above environmental standards (IS5) have detectable incidences of HYP. If sensitivity to HYP is population-specific, then increase in HYP from 5% in 1988 to >50% in 2005 (Grütjen et al. 2013) in some sites of the North Sea and its association with changes in genetic population structure should be evaluated.

Age and contaminant exposure have recognized effects in dab liver health (Feist et al. 2004; Stentiford et al. 2010). However, the liver pathology model also revealed that population genetic structure had an important effect on how pathologies develop with individual age. It is important to remember that the liver pathology classification collapses an array of 35 indicators, thus representing a suite of metabolic responses. Old fish in the northern Irish Sea exhibited consistently lower prevalence of neoplastic processes than their North Sea counterparts. Although such differences could be due to other contaminants or environmental parameters not accounted for here, it would be worthwhile investigating if the Liverpool Bay population–specific lower propensity to develop neoplasms, or less likelihood of surviving with them, is genetically based. Furthermore, the lack of malignant neoplasms of above 5-year-old dab might indicate that dab either develop malignant neoplasms and are likely to disappear, or develop benign neoplasms and individuals survive to join the >5 year classes. Such a system might provide an informative model in neoplasm-gene association studies. Together, these observations indicate that inferences of contaminant exposure based on liver pathology of dab over 4 years of age may be biased by population-specific sensitivity. Liver pathology of dab under 4 years of age seemed to truly represent heavy metal exposure. Cadmium has carcinogenic properties (Waisberg et al. 2003), and high concentrations were associated with the early onset of neoplastic processes, typical of site NS1 (Cefas 2003; Stentiford et al. 2009), where higher concentrations of cadmium are found in sediments (Langston et al. 1999). The opposite relationship was found for zinc, where high concentrations were associated with healthy livers and lower prevalence of inflammatory diseases. Zinc has been found to have protective effects against cadmium toxicity in other fish species (Malekpouri et al. 2011) through normalization of cadmium-disrupted antioxidant enzymatic pathways (Banni et al. 2011). Such processes could prevent the development of cadmium-induced neoplastic processes in dab. However, the relationship between zinc and liver health may be spurious as liver zinc concentrations were negatively correlated with age, and hence, high zinc and healthy livers correspond to one-year-old dab. Whether such a pattern is due to older dab having greater demand for, and faster metabolization of zinc (Sun and Jeng 1999), or young dab with high zinc exposure suffer high zinc poisoning (Hattink et al. 2006) requires further testing.

Genetic markers not only assisted in the identification of populations, but also yielded insights about whether dab disease phenotypes were indicative of exposure at capture location. Within the North Sea, Dogger Bank samples (NS1) are characterized by a high prevalence of skin hyperpigmentation, ulcerations and frequent occurrence of neoplastic processes (Cefas 2003; Stentiford et al. 2009). The existence of at least weak differentiation (Fig. 3) between coastal and Dogger Bank dab suggests that the latter are unlikely to mix extensively with the former; in addition, the lack of significant genetic differentiation among cohorts (from juveniles to 5-year-old adults) within sites is compatible with life-long residency at sampling sites, a particularly important attribute, and underlying assumption, in biomonitoring programmes (Stentiford et al. 2010; Bergek et al. 2012). Combined, these two characteristics suggest that dab captured at Dogger Bank are representative of locally higher concentration of contaminants (Langston et al. 1999).

The low prevalence of diseases in the English Channel may be a direct result of the absence of older fish (>4 years) at these sites, which typically exhibit higher disease prevalence than younger fish elsewhere (Stentiford et al. 2010). Hence, although sites in the English Channel are genetically similar to the North Sea, they should not be used as reference for the North Sea in biomonitoring programmes unless age is accounted for. Temporal genetic changes, such as those reported in this study at the western English Channel (EC2; Fig. 2), may command special treatment in biomonitoring analysis, as the susceptibility of immigrant individuals may be different to that of resident individuals.

Although contaminants have been shown to be capable of impacting neutral genetic diversity (Bourret et al. 2008; Nowak et al. 2009), we found no correlation between any of the population or individual measures of genetic diversity and either the biomarkers or contaminants (data not shown). The absence of a relationship between neutral heterozygosity and biomarkers/contaminants is not necessarily indicative of a lack of impact of contaminants on genetic diversity: contaminant effects may be associated with the frequency of alleles at non-neutral genes (Maes et al. 2005), or the balance between selective pressures and immigration may not be sufficiently skewed to generate signatures of genome-wide inbreeding (Tsitrone et al. 2001).

### Conclusion and relevance for biomonitoring programmes

Overall, our data reveal the existence of significant genetic structuring in dab at the regional scale of sea basins. The congruence among microsatellite markers and the observed temporal stability of genetic differentiation re-emphasizes the importance of temporal replicate sampling in elucidating genetic structuring among marine populations. The subtle differentiation found here may not necessarily imply high levels of gene flow among populations (Hauser and Carvalho 2008). On the contrary, the existence of low but significant differentiation values with neutral markers despite such large populations, and recent colonization history, suggests the existence of biologically relevant divergence associated with locally adapted populations. Furthermore, our study shows how genetically based structuring, together with other habitat variables, may be associated with population-specific heterogeneity in the nature and extent of response to contaminants and other environmental stresses between basins and, hence, is of utmost importance in the interpretation of biomonitoring data. Such genetic heterogeneity in marine fish across locales adds to increasing evidence that despite the traditional expectation of low divergence in potentially high gene flow marine taxa, that local adaptations exist across populations at remarkably small geographical scales (Larsen et al. 2007, 2012; Williams and Oleksiak 2008; Limborg et al. 2012; Hemmer-Hansen et al. 2013). It is important to emphasize that the population-specific variation in response found here does not necessarily emerge from adaptation to anthropogenic contaminant exposure, but may be the result of genetic drift, a by-product of adaptation to other selective pressures, or adaptation to naturally high levels of the elements involved in contamination. Although our results do not allow us to clearly disentangle the impacts of genes versus environment, they do provide a population framework that facilitates interpretation of biomonitoring results and the construction of testable hypothesis to further elucidate key drivers. To unveil the mechanisms behind the *heterogeneity of population-specific responses* would require the application of high-throughput population genomic approaches targeting either candidate genes of known function or genomic regions under selection combined with translocation/common garden and exposure experiments (Stinchcombe and Hoekstra 2008; Larsen et al. 2012). *Potential for adaptation* can be evaluated experimentally by analysing candidate genes on multigenerational selected lines (van Wijk et al. 2013). However, detection of *unequivocal adaptation to contaminant exposure in the wild* would require the assessment of multigenerational changes at known functional genes, pre- and postexposure, while ratifying no population replacement (Hansen et al. 2012).

Our study illustrates how genetic markers can elucidate population structure in a key biomonitoring species, delineating boundaries of comparable sampling sites and potential for differential response to environmental stress. It is recommended that biomonitoring programmes integrate the extent and spatiotemporal patterns of genetic structuring within assessments: individuals from target polluted sites should be compared to individuals from reference sites *within* the same population boundaries. In doing so, environmental managers would avoid confounding variance potentially due to population-specific inherent susceptibility, or alternatively, resistance to disease, and that which is truly symptomatic of exposure to contaminants. Additionally, population-specific disease-frequency baselines can then be established with evolutionary meaningful geographical boundaries, allowing detection of finer variation in spatial patterns of disease prevalence. Population genetics, biomonitoring and functional genomic analyses should be integrated to further understand the underlying role of genetic variation in biomarker prevalence and heterogeneity in measures of exposure (Cerdá et al. 2010). Ultimately, the incorporation routinely of population genetic approaches into biomonitoring and other programmes that assess ecosystem health enhances prospects for yielding ecologically meaningful predictive frameworks for assessing future response and options for remediation.

## Data archiving statement

Raw data for this study are available in Dryad: doi:10.5061/dryad.j7c74.

## Supporting Information

**Table S1.** Locus information.

**Table S2.** Genetic sample information.
