# Supplementary material for: Population genetics provides new insights into biomarker prevalence in dab (Limanda limanda L.): a key marine biomonitoring species
Source: Evol Appl. 2013 May 23;6(6):891–909. doi: 10.1111/eva.12074 (PMC3779091; doi:10.1111/eva.12074)
Supplement: Supplementary file 1 [file eva0006-0891-SD1.docx]

Table S1: Locus information: Locus= locus name; Na = Number of alleles at locus; Range = range of allele sizes (bp); ĤO = mean observed heterozygosity at locus; ĤE= mean expected heterozygosity at locus; *P*-value of HWE conformity= probability that the locus is in Hardy-Weinberg equilibrium expectations; *P*-value of genic differentiation = probability that alleles found at the locus are randomly distributed across samples; *F_IS_* = locus-specific inbreeding coefficient within individuals relative to the sample. *G’_ST_* = fixation index of alleles within samples relative to the total population; *G’’_ST_*= standardised fixation index.

Significant values after multiple testing correction are in **bold**.

Table S2: Genetic sample information: sampling site; CSEMP station number; Latitude (Lat.); Longitude (Long.); Sample Size (number of individual fish genotyped) sorted by collection year; Na = number of alleles encountered across all markers in every samples; *H_O_* = observed heterozygosity; *H_S_* = average within population heterozygosity; *F_IS_* = inbreeding coefficient; *P*-value HWE: probability of a sample conforming to Hardy Weinberg expectations for all loci with no null alleles.

Significant values after multiple testing correction are in **bold**.
